# Complete mitochondrial genome of the least weasel *Mustela nivalis* (Mustelidae) in Korea

**DOI:** 10.1080/23802359.2017.1372723

**Published:** 2017-10-17

**Authors:** Sang Jin Lim, Hye Ri Kim, Jae Youl Cho, Yung Chul Park

**Affiliations:** a Department of Forest and Environmental System, College of Forest & Environmental Sciences, Kangwon National University, Chuncheon, Republic of Korea;; b Ecosystem Research Division, National Park Research Institute, Wonju, Republic of Korea;; c Department of Genetic Engineering, Sungkyunkwan University, Suwon, Republic of Korea;; d Division of Forest Science, College of Forest & Environmental Sciences, Kangwon National University, Chuncheon, Republic of Korea

**Keywords:** Mitogenome, least weasel, *Mustela nivalis*, Mustelidae

## Abstract

The complete mitogenome (MF459691) of *Mustela nivalis* contains total 16,502 bp in length and consists of a control region and a conserved set of 37 genes. The phylogenetic tree of the family Mustelidae constructed using 18 mitogenome sequences from 16 mustelid species of 6 genera shows that the mustelid species are separated into two main groups. All the members of the genus *Martes* form a monophyletic group, which has the genus *Melogale* as sister clade, and the clade of the two genera is sister to that of the genera *Enhydra* and *Lutra*. Among species of the genus *Mustela*, with the exclusion of *M. frenata* with *Neovison vison* as sister species, all the other species of the genus *Mustela* are well grouped. All members of *M. nivalis* are well placed within the species *M. nivalis* clade with the clade of *M. itatsi* and *M. sibirica* as sister group.

The least weasel *Mustela nivalis* is the smallest carnivorous predator of the genus *Mustela* and is widely distributed throughout Holarctic region, taking in much of Europe, northern Asian and northern North America (Sheffield and King 1994; Abramov and Baryshnikov 2000; McDonald et al. 2016). Despite its wide distribution and presumed large population in worldwide level (McDonald et al. 2016), this species was listed as endangered species in South Korea in view of decrease with population size in fragmented forests.

We characterized the complete mitogenome of a *M. nivalis* individual in Korea. A fresh tissue for genomic DNA extraction was collected from the road-killed individual in agroecosystem in Odaesan National Park (N37 42 45.1 E128 36 00.6). The voucher specimen (MUMUNI-1) was deposited in the Wildlife and Fish Conservation Center of the Institute of Forest Science, Kangwon National University. Genomic DNA extraction, PCR and genome annotation were conducted according to the previous studies (Yoon et al. 2013; Jeon and Park 2015). A previously published mitogenome of *M. nivalis* (NC_020639) was used as a reference for gene annotation and primer design.

The complete mitogenome (MF459691) of *M. nivalis* contains total 16,502 bp long, which consists of a control region and a conserved set of 37 genes including 13 protein-coding genes (PCGs), 22 tRNA genes, and two ribosomal RNA genes (*12S rRNA* and *16S rRNA*). The majority of 13 PCGs (10 of 13 PCGs) use ATG as start codon, whereas *Nd3*and *Nd5* initiate with ATT and *Nd2* starts with ATC. Of the 13 PCGs, the incomplete stop codons (T– or TA–) are used for termination of *Nd1, Nd2, Cox3*, and *Nd4* (T–) and *Nd3* (TA–). ATT and AGA are used as stop codons in *Nd6* and *Cytb*, respectively, and the other six genes end with TAA. The 22 tRNA genes range from 62 bp (*tRNA^Ser(AGY)^*) to 75 bp (*tRNA^Leu(UUR)^*) in length. Lengths of the two rRNA genes and control region are 1009 bp (*12S rRNA*), 1572 bp (*16S rRNA*), and 1016 bp (control regions), respectively. The replication origin *O_R_*, which is 35 bp long, is located between *tRNA^Asn^* and *tRNA^Cys^* within the WANCY tRNA cluster as seen in most vertebrates (Kim and Park 2012; Kim et al. 2013; Yoon et al. 2013).

According to the phylogenetic analysis of the family Mustelidae (Figure 1), the mustelid species were separated into two main groups. In Group 1, all the members of the genus *Martes* form a monophyletic group, which has the genus *Melogale* as sister clade, and the clade of the two genera is sister to that of the genera *Enhydra* and *Lutra*. In Group 2, with the exclusion of *M. frenata* with *Neovison vison* as sister species, all the other species of the genus *Mustela* are well grouped. All members of *M. nivalis* are well placed within the *M. nivalis* clade with the clade of *M. itatsi* and *M. sibirica* as sister group.

**Figure 1.**
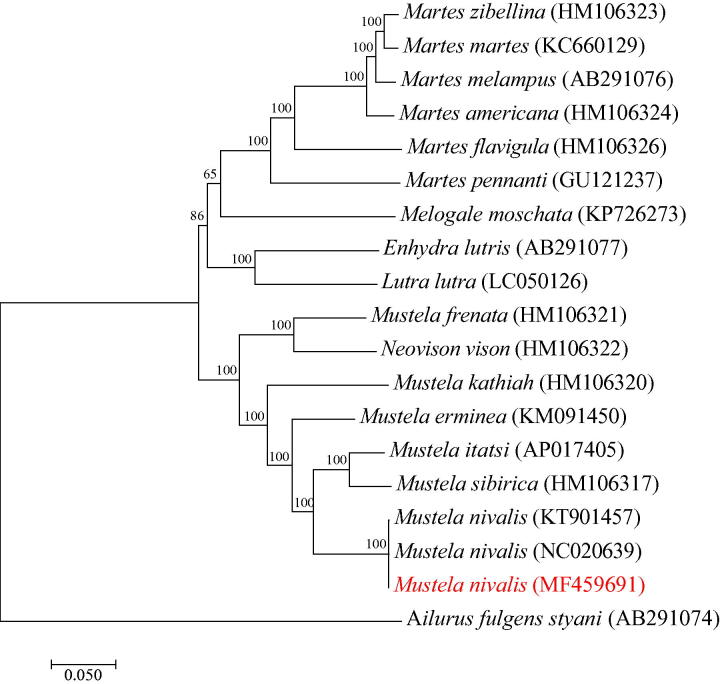
The phylogenetic tree of the family Mustelidae constructed using the maximum-likelihood (ML) procedures implemented in MEGA7 (Kumar et al. 2016). The phylogenetic relationship was constructed based on 18 mitogenome sequences known to date from 16 mustelid species in six genera. The ML tree was generated using the GTR + G+I model, and the robustness of the tree was tested with 1000 bootstrap. The numbers on the branches indicate bootstrap values. The complete mtDNA of *Mustela nivalis* (MF459691) was obtained from this study.
